# Neuronal *Lamin* regulates motor circuit integrity and controls motor function and lifespan

**DOI:** 10.15698/cst2018.09.152

**Published:** 2018-08-17

**Authors:** Lisa J. Oyston, Yong Qi Lin, Thang M. Khuong, Qiao-Ping Wang, Man Tat Lau, Teleri Clark, G. Gregory Neely

**Affiliations:** 1 The Dr. John and Anne Chong Lab for Functional Genomics, Charles Perkins Centre and School of Life and Environmental Sciences, The University of Sydney NSW 2006, Australia.; 2 Neuroscience Division, Garvan Institute of Medical Research, 384 Victoria Street, Darlinghurst, Sydney, NSW 2010, Australia.; 3 St Vincent’s Clinical School, Faculty of Medicine, UNSW Sydney NSW 2052, Australia.

**Keywords:** aging, Lamin, dopaminergic neurons, neurodegeneration, Drosophila, synapse

## Abstract

Neuronal aging involves a progressive decline in cognitive abilities and loss of motor function. Mutations in human *Lamin* genes (*LMNA, LMNB1, LMNB2*) lead to a wide-range of diseases including muscular dystrophy, peripheral neuropathy and progeria. Here we investigate the role of neuronal* Lamin* in regulating age-related phenotypes. Neuronal targeting of *Lamin* led to shortened lifespan, progressive impairment of motor function and loss of dopaminergic (DA) neurons within the protocerebral anterior medial (PAM) cluster in the *Drosophila* *melanogaster *brain. Loss of neuronal *Lamin* caused an age-related decline in neural physiology, with slower neurotransmission and increased chance of motor circuit failure with age. Unexpectedly, *Lamin*-dependent decline in motor function was specific for the chemical synapses of the dorsal longitudinal muscle (DLM). Together these findings highlight a central role for *Lamin* dysfunction in regulating neuronal survival and motor circuit physiology during aging.

## INTRODUCTION

Nuclear architecture and nuclear membrane function is a critical regulator of the aging process 1. The nuclear lamina is a filamentous network that lines the inner nuclear membrane, providing a structural scaffold for the nucleus. This network is made up of type V intermediate filaments known as lamins that tether protein and chromatin complexes to the inner nuclear membrane. Mammals possess three lamin genes, an A-type gene, *LMNA* which encodes the alternatively spliced variants lamin A and lamin C, as well as two B-type genes, *LMNB1* and *LMNB2* 2. Mutations in human *Lamin* genes (*LMNA*, *LMNB1*, *LMNB2*) cause a range of severe disorders termed laminopathies, which include muscular dystrophies, peripheral neuropathies and progeria, an accelerated aging syndrome 2. Alterations in the nuclear lamina have also been implicated in normal aging, suggesting that nuclear integrity and genomic stability are critical for cellular health throughout life 3.

B-type lamins are constitutively and ubiquitously expressed whilst A-type lamins show more specific expression patterns in differentiated cells 4. Differential expression patterns of A- and B-type lamins may explain the tissue-specific pathology that occurs in many laminopathies, for example, the absence of dementia in Hutchinson-Gilford progeria patients, which is caused by mutations in *LMNA*.

*Drosophila *are the only invertebrates known to possess both A (*Lamin C*) and B-type lamins (*Lamin Dm0*; referred to as *Lamin* throughout this publication) 5. Both *Drosophila* and vertebrate lamins evolved from a single gene in a common ancestor and possess similar expression patterns and molecular motifs 5,6.

The role of the nuclear lamina within the adult brain is somewhat unclear.* Lamin* is the only nuclear lamin found to be expressed in the nervous system 7,8. Even in *Lamin* null mutant flies, *Lamin C* is not detectable, suggesting limited compensation within this compartment 7,8. B-type *Lamin* genes (*Lmnb1*, *Lmnb2*) are essential for neuronal survival and migration 9, while *Lamin* expression decreases in the aging *Drosophila* brain 10. Moreover, transgenic expression of *Tau* causes a decrease in lamin protein, and a *Lamin* partial loss of function mutation significantly reduces lifespan and promotes neuronal apoptosis 11. Here, we investigate the role of *Lamin* on neurological function within the aging *Drosophila* brain.

## RESULTS 

**Figure 1:**
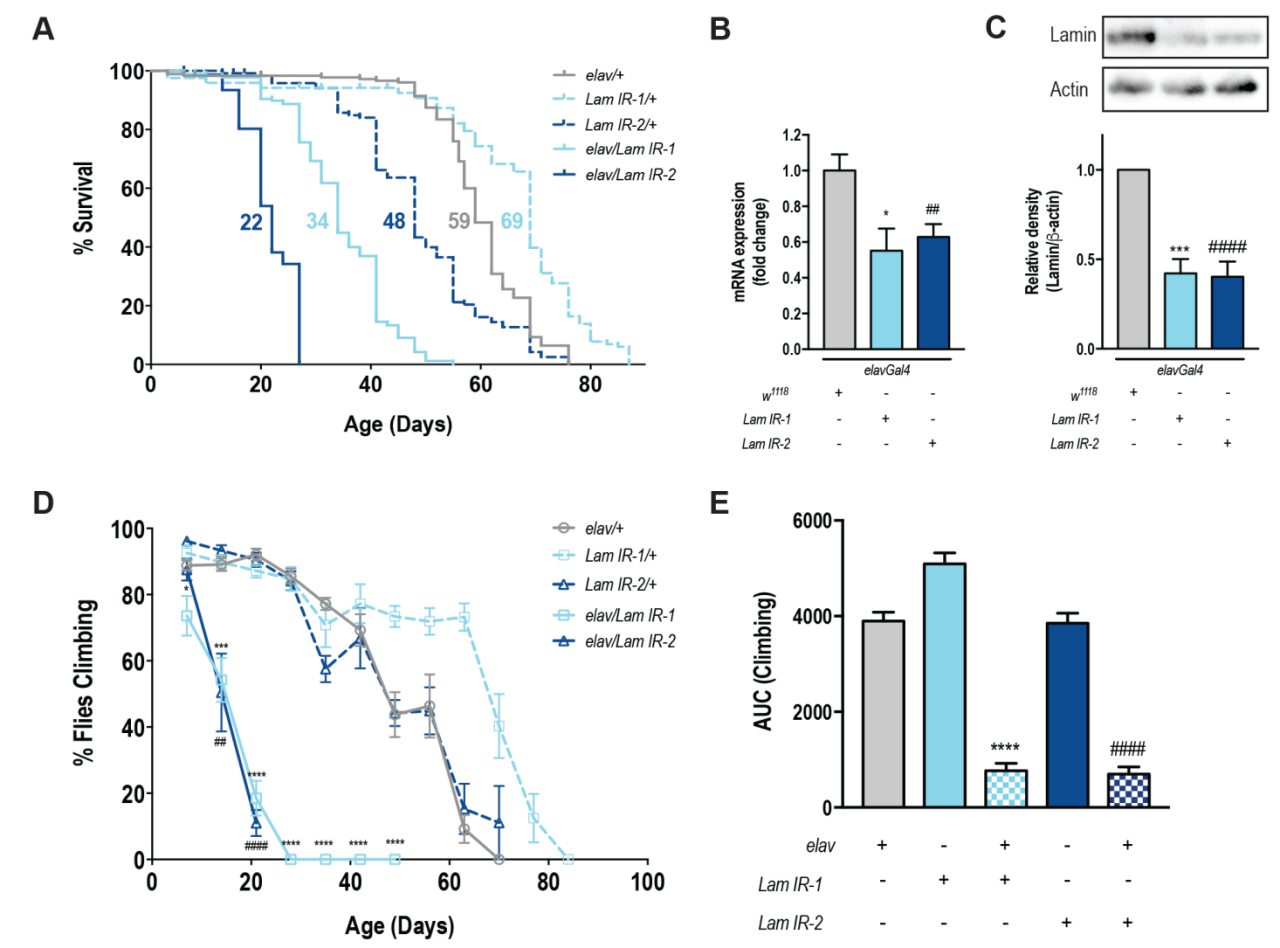
FIGURE 1: Neuronal knockdown of *Lamin* causes decreased lifespan and locomotor ability. **(A)** Pan neuronal knockdown of *Lamin (elav/Lam IR*) causes a significant decrease in lifespan compared to control flies (*elav/+*). **(B)** qPCR for knockdown efficiency of *Lamin *RNAi hairpins showed a significant decrease in *Lamin* mRNA expression for both hairpins. **(C)** Western blot of fly heads showed a significant decrease in *Lamin* protein expression with *Lam IR-1* and *Lam IR-2* knockdown. n=5. **(D-E)** Locomotor ability is significantly impaired in *elav/Lam IR* flies. n(50 animals per group. Data represents mean ( SEM. Climbing, western blot and qRT-PCR data was analysed using one-way ANOVA with post-hoc Dunnett test for multiple comparisons. Lifespan analysis was done using a log-rank (Mantel-Cox test). *comparison between *elav/+* and *elav/Lam IR-1*. #comparison between *elav/+* and *elav/Lam IR-2*. *p<0.05; **p<0.01; ***p<0.001; ****p<0.0001. *elav/+ = elavGal4; +/+; UAS-mCD8GFP > w^1118^; Lam IR/+ = w^1118^ > Lam IR; elav/Lam IR = elavGal4; +/+; UAS-mCD8GFP > Lam IR*.

To specifically assess the role of *Lamin* within the nervous system, we used a tissue-specific transgenic RNAi approach 12. *elavGal4* drives transgenic GFP expression *(UAS-mCD8GFP*) across the fly brain (**Fig S1A-F**) and these flies showed no difference in lifespan or motor function when compared to *elavGal4* alone (**Fig S1G-H**). Neuronal knockdown of *Lamin* with two independent RNAi lines showed a significant reduction in lifespan (**Fig 1A, Fig S1I-K**). For each RNAi line, neuronal knockdown of *Lamin* was ~50% of control levels by qPCR (**Fig 1B**), and western blot of heads from *elav/Lam IR* flies also showed a ~60% reduction in Lamin protein levels (**Fig 1C**). Functionally, a decrease in neuronal *Lamin* led to a rapid and progressive loss of motor function over the first 21 days of life (**Fig 1D**) and overall, neuronal *Lamin* caused an ~80% decline in climbing ability over the fly’s lifespan (**Fig 1E**). Taken together, decreased expression of *Lamin* within the nervous system is sufficient to shorten lifespan and impair motor function with age.

**Figure 2:**
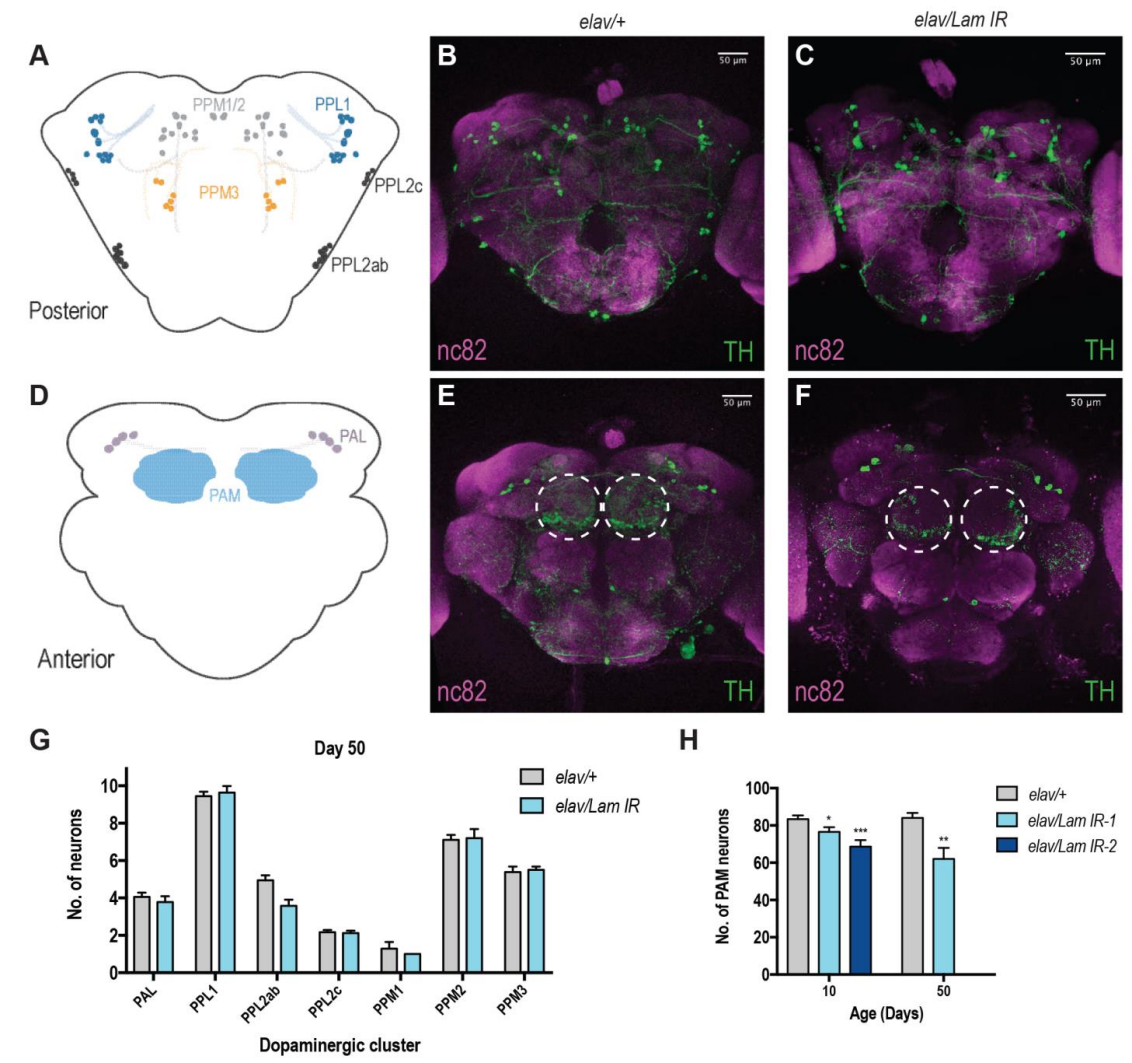
FIGURE 2: Neuronal knockdown of *Lamin *causes an age-dependent loss of PAM cluster dopaminergic neurons. **(A) **Schematic of the posterior dopaminergic neurons of the *Drosophila* brain. Representative images of posterior dopaminergic neuron clusters in **(B) ***elav/+* and** (C) ***elav/Lam IR* flies at 50 days old. **(D) **Schematic of the anterior dopaminergic neurons of the* Drosophila* brain. Representative images of the anterior dopaminergic neuron clusters in** (E) ***elav/+* and** (F) ***elav/Lam IR* flies at 50 days old.** G **Quantification of dopaminergic neuron number in the posterior and anterior clusters of the fly brain at 50 days.** (H) **Quantification of dopaminergic neurons within the PAM cluster of the fly brain. n(7 animals per group. Data represents mean ( SEM and was analysed using student’s *t*-test. *p<0.05; **p<0.01; ***p<0.001. *elav/+ = elavGal4; +/+; UAS-mCD8GFP > w^1118^; elav/Lam IR = elavGal4; +/+; UAS-mCD8GFP > Lam IR.*

DA neurons are known to be responsible for locomotor decision making and fine tuning in vertebrates and invertebrates 13 and are well characterised within the fly brain 14,15. Decline in motor function has also been associated with progressive loss of DA neurons with age 16. Moreover, in *Drosophila*, it has been shown that neurons within the PAM cluster on the anterior side of the fly brain are responsible for startle-induced locomotor function (**Fig 2D**) 17. Neuronal *Lamin* knockdown animals displayed no apparent loss of DA neurons across most areas of the brain (**Fig 2A-C, G, S2A-D, I**), however we did observe a progressive loss of DA neurons within the PAM cluster (**Fig 2D-F, S2E-H**), and this effect was most pronounced in aged animals (**Fig 2H**). Assessment of DA neurons in 50-day old *elav/Lam IR-2* flies was not possible due to their maximum lifespan of 27 days (**Fig 1A**). However, loss of PAM DA neu-rons was observed in *elav/Lam IR-2* flies at 10 days of age (**Fig 2H**), suggesting fly lifespan may be correlated to DA neuron loss in the PAM cluster. Together our results suggest that neuronal *Lamin* levels are critical for the brain during aging, and dysregulation of *Lamin* expression can lead to degeneration of DA neurons.

Due to the profound effects of neuronal-specific *Lamin* knockdown on age-related behaviours (climbing, lifespan and neurodegeneration), we decided to investigate its effects on neuronal function. Age-related deficits in *Drosophila *lifespan and climbing behaviors manifest in the functional decline of the fly’s giant fiber system (GFS) 18, a well-defined neuronal circuit that controls jump and flight responses 19,20.

**Figure 3:**
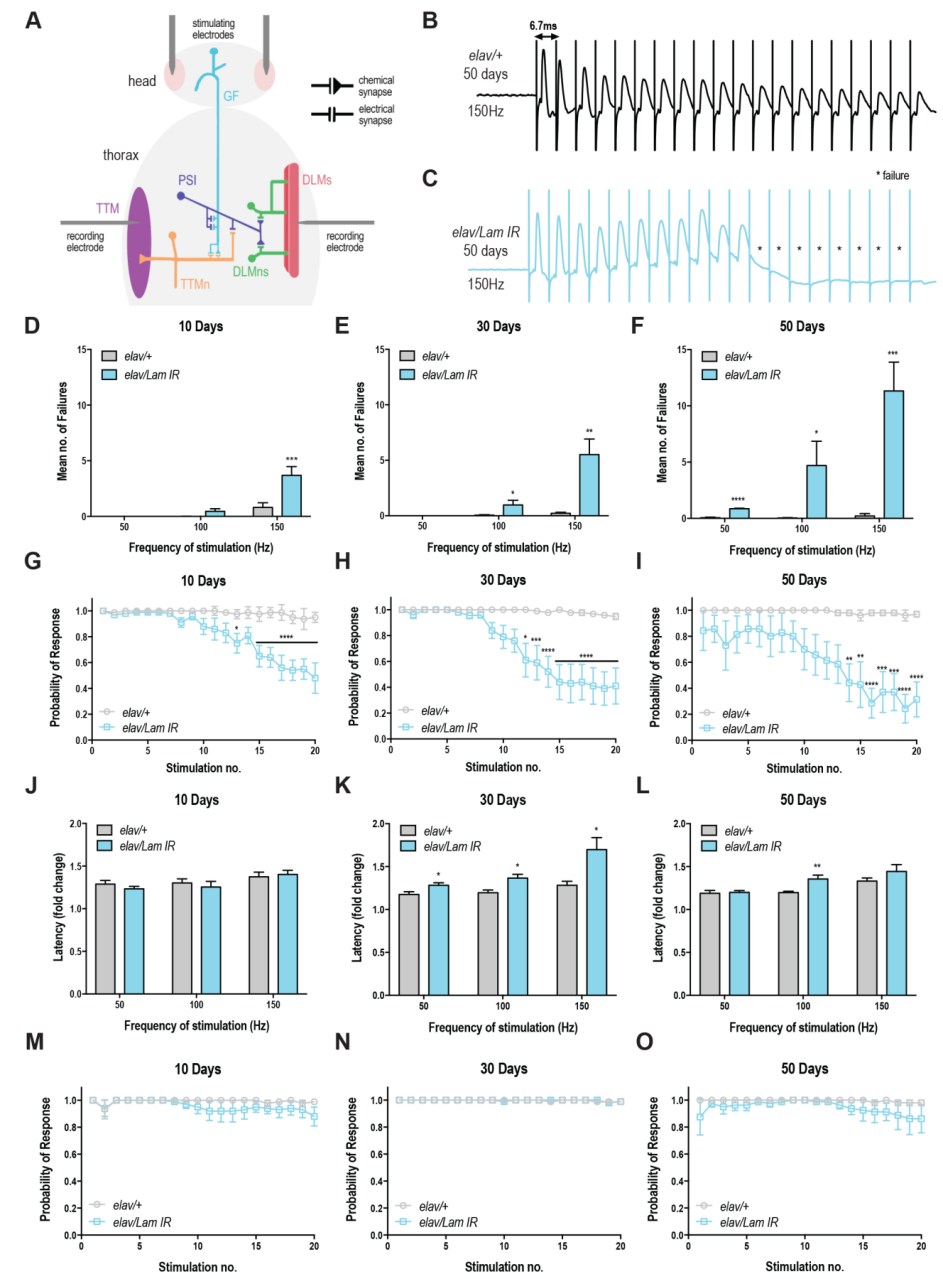
FIGURE: 3 Neuronal knockdown of *Lamin* causes increased failure and latency of the DLM. **(A) **Schematic of experimental setup for recording from the GFS. TTM = tergotrochanteral muscle; TTMn = tergotrochanteral motorneuron; PSI= peripherally synapsing interneuron; DLM = dorsal longitudinal muscle; DLMNn = dorsal longitudinal motorneuron. Representative traces from the DLM in **(B)** *elav/+ *and **(C)** *Lamin* knockdown flies (*elav/Lam IR*) aged 50 days, stimulated at 150 Hz. Asterisks indicate failure to respond. Mean number of failures for *elav/+* and *Lamin* knockdown flies (*elav/Lam IR*) at **(D) **10 days, **(E)** 30 days and **(F)** 50 days old. **(G-I) **The probability of DLM response in *Lamin* knockdown flies (*elav/Lam IR*) is significantly decreased after repeated stimulations, in an age-dependent manner. **(J)** No significant difference in latency was found in young flies (10 days) with *Lamin* knockdown (*elav/Lam IR*) when compared to *elav/+*. **(K-L)** Aged flies (30 days and 50 days) show a significant increase in DLM latency with *Lamin* knockdown (*elav/Lam IR*). **(M-O) **The probability of TTM response in *Lamin* knockdown flies (*elav/Lam IR*) shows no significant difference from *elav/+*. n(7 animals per group. Data are mean ( SEM. Data was analysed using two-way ANOVA with Bonferroni test for multiple comparisons. Response latency was analysed using multiple t-tests with Holdam-Sidak correction for multiple comparisons. *p<0.05; **p<0.01; ***p<0.001; ****p<0.0001. *elav/+ = elavGal4; +/+; UAS-mCD8GFP > w^1118^; elav/Lam IR = elavGal4; +/+; UAS-mCD8GFP > Lam IR.*

To assess neurotransmission in the GFS, we recorded from the DLM, the indirect flight muscle which is controlled via a glutamatergic synapse from the peripherally synapsing interneuron (PSI; **Fig 3A**) 20. Compared to control animals, *Lamin* knockdown animals showed a progressive loss of circuit function, manifesting in response failures on repeated train stimulation (**Fig 3B-C**). Quantification of these results highlights the loss of motor circuit function over time (**Fig 3D-F**) with maximal failures (11.32 ( 2.57) observed in 50-day old flies with neuronal *Lamin* knockdown at 150 Hz (**Fig 3F**). Control flies across all ages and all stimulation frequencies showed <1 mean failures (**Fig 3D-F**). Qualitatively, the probability of response for *Lamin* knockdown animals declined on repeated stimulation at each age point (**Fig 3G-I**), however, 50-day old animals showed baseline defects in circuit responses even without repeated stimulation, highlighting the extreme age-related loss of motor circuit function in these flies. No difference was observed in DLM response latency at 10 days in *Lamin* knockdown or control flies (**Fig 3J**). Moreover, DLM response latency was significantly increased in *Lamin *knockdown flies at 30 days of age (**Fig 3K**) compared to control. This effect could arise from direct loss of circuit or neuromuscular junction integrity, or indirectly via trans-synaptic effects that alter the responsiveness of the post-synaptic muscle. A significant difference in latency was also observed at 50 days and 100 Hz stimulation, however due to the 70-75% failure rate at the 20^th^ pulse in 50-day-old *Lamin* knockdown flies stimulated at 150 Hz, only a few latency values could be calculated and no significant difference was observed in this case (**Fig 3L**). Together, we show that neuronal-specific loss of *Lamin* triggers a severe age-related decline in motor circuit performance.

Recordings were also taken from the tergotrochanteral muscle (TTM), which controls the "jump" phase of the fly’s escape response via mixed electrochemical synapses 20. Unexpectedly, no significant increase in failure rate (**Fig S3A-E**), decrease in response probability (**Fig 3M-O**), or change in circuit latency (**Fig S3F-H**) was observed. Together, these data show that DLM synapses are sensitive to changes in the nuclear lamina.

## DISCUSSION

*Drosophila* has been an essential model organism in the discovery of the plasticity of aging, helping to identify genetic pathways and biochemical processes capable of influencing the rate of aging 21,22. Here we expand on previous findings 11, to show targeting neuronal *Lamin* causes a progressive loss of locomotor function and decreased lifespan. These results are consistent with previous data on* Lamin* null and partial loss of function mutants that have been shown to be lethal or semi-lethal with surviving adults experiencing locomotor deficits and decreased lifespan 7,8,11,23. Studies of neuronal *Lamin* knockdown previously conducted by Frost *et al*. (2016) also showed comparable locomotor dysfunction to our study in the absence of decreased lifespan 11. This discrepancy may be due to differences in genetic background, diet or environment and further investigation is required.

Defects in negative geotaxis behaviour have previously been accompanied by age-dependent loss or decreased tyrosine hydroxylase immunoreactivity in *Drosophila *DA neurons 24,25,26,27,28. Here we show that neuronal *Lamin* knockdown is sufficient to cause age-dependent loss of DA neurons within the PAM cluster, however, the mechanism behind this loss remains unclear. *Lamin* dysfunction has previously been shown to cause apoptotic neuron death as a result of genomic architecture disruption, DNA damage and cell-cycle reactivation 11. Thus, selective DA neurodegeneration in *Lamin* knockdown flies may occur due to increased susceptibility of PAM cluster neurons to age-related disruptions in nuclear envelope integrity, ultimately resulting in apoptosis. Behaviourally, DA neurodegeneration may then lead to decreased locomotor function and shortened lifespan.

Of note, we observed enhanced survival and climbing in *Lam IR-1/+* lines when compared to the *elav/+* or *Lam IR-2/+* control groups. Based on these results, we cannot determine if *Lam IR-1/+* flies exhibit enhanced health or if *elav/+* and *Lam IR-2/+* animals show a relative decrease in lifespan and motor function. Factors that could mediate this effect include an overall weakness of *elav/+* animals due to *gal4* expression, or a non-specific leakiness of the *Lam IR-2 *transgene which confers a disadvantage to this line*. *Alternatively, as these lines have been created a number of years ago, it is possible that secondary unknown mutations affect the health of one or more lines. Regardless, *elav/Lam IR* animals showed a 50% reduction in lifespan compared to either parental control consistent with a specific role for *Lamin* in promoting neuronal health.

In this study, we have shown an age-dependent decrease in conduction velocity and an elevated failure rate at the *Drosophila* DLM following decreased *Lamin *expression. Martinez *et al*. (2007) have previously shown the effects of aging on neuronal function within the GFS, showing an association between aging and the number of successful DLM responses to high frequency stimulation in wild-type flies (100-200 Hz). In their study, elevated failure rate of the DLM muscle occurred in 35-45 days old animals stimulated above 150 Hz 18. In our study, we did not observe failures in 50-day old wild type animals, however our maximum stimulation was 150 Hz, and thus a lack of failure at this frequency is consistent with the findings of Martinez *et al*. (2007). We have shown a progressive increase in DLM failure rate in *Lamin* knockdown flies starting at day 10 and these animals exhibit dramatically reduced lifespan. The synaptic impairment of the DLM response and shortened lifespan induced by decreased *Lamin* expression are reminiscent of *Drosophila* models of motor neuron disease 29,30, suggesting that neurodegeneration may be responsible for the phenotypes observed here.

We also observed that TTM latency and failure rate were unaffected by *Lamin* knockdown. The DLMn is pre-dominantly activated by chemical glutamatergic synapses; the TTMn on the other hand is predominantly activated by electrical gap junctions directly from the GF 20. The differences in response between the compound action potentials could thus be explained by the fact that *Lamin* defects are specific to chemical synapses rather than electrical gap junctions 31. Alternatively, RNAi efficiency may differ between TTM and DLM circuits, which could explain why TTM responses were independent of *Lamin*. Neuromuscular junction defects have also been reported in *Drosophila Lamin C* mutants, caused by disruptions to nuclear budding leading to improper differentiation of synaptic boutons 32. Together these results suggest that *Lamin *dysfunction at neuromuscular junctions may be behind the manifestation of some laminopathies (muscular dystrophies) and Parkinson’s disease?-like phenotypes.

Overall, we show here that neuronal-specific *Lamin *is a critical regulator of lifespan, neurodegeneration, and motor circuit health, and dysregulation of neuronal *Lamin* expression/function may contribute to multiple pathologies associated with aging and age-related diseases.

## MATERIALS AND METHODS

### Fly stocks

Flies were maintained on a standard diet of agar, corn meal, yeast and molasses at 25°C, 65% humidity under a 12h:12h light:dark cycle. e*lavGal4[C155] *and *UAS-mCD8GFP* were obtained from the Bloomington Drosophila Stock Center. The *Lamin* RNAi hairpins (107419 and 45635) and *w^1118^* were obtained from the Vienna Drosophila Resource Centre’s knockdown libraries (Dietzl et al., 2007). An isogenic *w^1118^* background was maintained throughout all experiments.

### Lifespan assay

For assessment of fly lifespan, 3 vials of 15 male flies were collected per genotype and the number of dead flies was counted every 2-3 days when flies were transferred to new medium. This experimental paradigm was repeated at least three times.

### Climbing assay

Locomotor function was assessed once per week throughout the fly’s lifespan, as previously described 33,34. Briefly, flies were gently tapped to the bottom of the vial and given 10 seconds to climb a distance of 5 centimeters. The number of flies that failed to reach the distance was then recorded. Each vial was tested three times per time point with 30 seconds rest in between trials.

### qRT-PCR for knockdown efficiency

RNA was extracted from ~30 heads of 10-day-old male flies using Purezol (Bio-Rad Laboratories). All extracted RNA samples met the quality for qPCR (A260/A280>2.0, A260/A230>1.8). After quantification, 1 mu;g of RNA was reverse transcribed into cDNA using the iScript cDNA synthesis kit (Bio-Rad Laboratories). qRT-PCR was performed using a Sensimix probe and Syto 9 dye as per the manufacturer’s instructions (Bioline) and run on a LightCycler 480 Instrument II (Roche). Cycling conditions were as follows:

1. Initial denaturation: 95°C for 10 minutes

2. PCR cycling: 95°C for 15 seconds, 55°C for 15 seconds, 72°C for 15 seconds

Repeat for 45 cycles

3. Melting curve analysis

Gene expression was normalised to the reference gene *rp49* and relative mRNA abundance was calculated by the ((CT method. Primers were designed for *Lamin* (forward: CCACTGCTGAGGGCAATGTC; reverse: TTCCAGGTTCTTCCGCGTTT) and *rp49* (forward: CGGATCGATATGCTAAGCTGT; reverse: GCGCTTGTTCGATCCGTA) using PrimerBlast (NIH).

### Western blot

30 *Drosophila* heads from 10-day-old male flies were homogenised for each genotype using a motorised pestle in 60 mu;L of 1 x Laemmli loading buffer. Homogenised samples were then boiled at 100°C for 5 minutes and loaded onto a 10% SDS-page gel. Proteins were separated by electrophoresis at a constant voltage of 120 V for 90 minutes. Precision Plus Protein WesternC pre-stained molecular weight standards (Bio-Rad Laboratories) were loaded onto the gels for protein size comparisons. Gels were transferred onto a PVDF (Bio-Rad Laboratories) membrane at 1.0 A, 25 V for 30 minutes, using the Trans-Blot Turbo system (Bio-Rad Laboratories). Immunoblots were blocked in 5% non-fat milk in TBS (0.8% NaCl, 0.2% Tris, pH 7.6) for 30 minutes and then incubated with (-actin (ab8227; 1:2000; Abcam) and Lamin (#AL67.10; 1:50; Developmental Studies Hybridoma Bank) primary antibodies for 24 hours at 4°C. Blots were then washed 3 times with TBST (0.8% NaCl, 0.2% Tris, 0.1% Tween-20) and incubated with the appropriate secondary antibodies for 1 hour at room temperature. Washing steps were then repeated before blot development and exposure on a Chemidoc XRS+ (Bio-Rad Laboratories) for visualisation. ImageLab software (Bio-Rad Laboratories) was used for band molecular weight and densitometry analysis.

### Brain dissection and dopaminergic neuron staining and quantification

Adult brains from male flies were dissected and stained for tyrosine hydroxylase (TH) as previously described 35. Briefly, brains were dissected in ice-cold HL-3 solution (70 mM NaCl, 5 mM KCl, 10 mM NaHCO_3_, 5 mM HEPES, 115 mM sucrose, 5 mM trehalose, 20 mM MgCl_2_, pH 7.2) and fixed in 4% paraformaldehyde (PFA)/ phosphate-buffered saline with 0.4% Triton X-100 (PBT) solution for 20 minutes at room temperature. Fixed brains were then washed twice with quick inversion and then three times for 20 minutes at room temperature, using PBT. Brains were blocked with 5% normal goat serum in PBT for 30 minutes at room temperature. Blocked brains were then stained with primary antibodies; anti-TH (1:100; Millipore #AB152) and anti-nc82 (1:75; Developmental Studies Hybridoma Bank) for two nights at 4°C and then washed as above. The secondary antibodies goat anti-mouse Alexa 555 and goat anti-rabbit Alexa 647 (1:500; Invitrogen Molecular Probes) were then applied for two nights at 4°C. Stained and dissected brains were then washed again and mounted in Vectashield (Vector Laboratories) as previously described 27. Confocal stacks of brains were acquired using a Leica SP8X confocal microscope and used to quantify the number of dopaminergic neurons present in the PAM, PAL, PPL1, PPL2, PPL3, PPM1, PPM2 clusters. The number of neurons was scored off blinded z-stacks using the ImageJ Cell Counter plugin. Maximum projections of the posterior and anterior orientations were also collected.

### Electrophysiological recordings from the GFS

GFS recordings were performed as described previously 19. Briefly, flies were anesthetised on ice and then transferred to a petri dish where fly wings and legs were mounted in dental wax, ventral side down. For stimulations and recordings from the TTM and DLM, five sharp tungsten electrodes were used: two for stimulating the GF, one as a reference electrode and two for recording from the TTM and DLM, respectively. High frequency train stimulations of 20 pulses were delivered to the GF at 50, 100 and 150 Hz. This process was repeated 10 times with 3-5 min of rest between stimulation trains. 0.2 Hz stimulations were used prior to each high frequency stimulation train to confirm that electrodes were still situated in the correct muscle. The probability of response, at a particular frequency of GF stimulation, due to a particular stimulus was calculated from the proportion of successful responses for both TTM and DLM pathways. Response latency was measured as the time between the stimulus artefact and the first detectable voltage deflection of the corresponding response. Latency fold change was calculated as the ratio of the 20^th^ pulse latency and the 1^st^ pulse latency of the stimulation train.

### Statistical methods

Data are represented as mean ± SEM. Climbing ability was analysed using one-way ANOVA with post-hoc Dunnett test or multiple t-tests with Holdam-Sidak correction. Lifespan was analysed using the log-rank Mantel-Cox test. qPCR and western blot data were analysed using one-way ANOVA with post-hoc Dunnett test. All other experiments were analysed using student’s *t*-test or two-way ANOVA with Bonferroni multiple comparisons test. Significance was considered at a *p*-value less than or equal to 0.05. All statistical analysis was performed using GraphPad Prism 7.0.

## SUPPLEMENTAL MATERIAL

Click here for supplemental data file.

All supplemental data for this article are also available online at http://www.cell-stress.com/researcharticles/neuronal-lamin-regulates-motor-circuit-integrity-and-controls-motor-function-and-lifespan/.

## Abbreviations

DA: dopaminergic
DLM: dorsal longitudinal muscle
GFS: giant fiber system
PAM: protocerebral anterior medial
TTM: tergotrochanteral muscle

## References

[1] López-Otín C, Blasco MA, Partridge L, Serrano M, Kroemer G (2013). The hallmarks of aging.. Cell.

[2] Burke B, Stewart CL (2013). The nuclear lamins: Flexibility in function.. Nat Rev Mol Cell Biol.

[3] Scaffidi P, Misteli T (2006). Lamin A-dependent nuclear defects in human aging.. Science.

[4] Young SG, Jung HJ, Lee JM, Fong LG (2014). Nuclear lamins and neurobiology.. Mol Cell Biol.

[5] Melcer S, Gruenbaum Y, Krohne G (2007). Invertebrate lamins.. Exp Cell Res.

[6] Stuurman N, Heins S, Aebi U (1998). Nuclear Lamins: Their Structure, Assembly, and Interactions.. J Struct Biol.

[7] Osouda S, Nakamura Y, De Saint Phalle B, McConnell M, Horigome T, Sugiyama S, Fisher PA, Furukawa K (2005). Null mutants of Drosophila B-type lamin Dm0 show aberrant tissue differentiation rather than obvious nuclear shape distortion or specific defects during cell proliferation.. Dev Biol.

[8] Lenz-Böhme B, Wismar J, Fuchs S, Reifegerste R, Buchner E, Betz H, Schmitt B (1997). Insertional mutation of the Drosophila nuclear lamin Dm0 gene results in defective nuclear envelopes, clustering of nuclear pore complexes, and accumulation of annulate lamellae.. J Cell Biol.

[9] Coffinier C, Jung H-J, Nobumori C, Chang S, Tu Y, Barnes RH, Yoshinaga Y, de Jong PJ, Vergnes L, Reue K, Fong LG, Young SG (2011). Deficiencies in lamin B1 and lamin B2 cause neurodevelopmental defects and distinct nuclear shape abnormalities in neurons.. Mol Biol Cell.

[10] Tran JR, Chen H, Zheng X, Zheng Y (2016). Lamin in inflammation and aging.. Curr Opin Cell Biol.

[11] Frost B, Bardai FH, Feany MB (2016). Lamin Dysfunction Mediates Neurodegeneration in Tauopathies.. Curr Biol.

[12] Dietzl G, Chen D, Schnorrer F, Su K-C, Barinova Y, Fellner M, Gasser B, Kinsey K, Oppel S, Scheiblauer S, Couto A, Marra V, Keleman K, Dickson BJ (2007). A genome-wide transgenic RNAi library for conditional gene inactivation in Drosophila.. Nature.

[13] Beninger RJ (1983). The role of dopamine in locomotor activity and learning.. Brain Res.

[14] Mao Z, Davis RL (2009). Eight different types of dopaminergic neurons innervate the Drosophila mushroom body neuropil: anatomical and physiological heterogeneity.. Front Neural Circuits.

[15] White KE, Humphrey DM, Hirth F, Sweeney ST, Birman S, De CN (2010). The dopaminergic system in the aging brain of Drosophila.. Front Neurosci.

[16] Seidler RD, Bernard JA, Burutolu TB, Fling BW, Gordon MT, Gwin JT, Kwak Y, Lipps DB (2010). Motor control and Aging: Links to age-related brain structural, functional and biomechanical effects.. Neurosci Biobehav Rev.

[17] Riemensperger T, Issa AR, Pech U, Coulom H, Nguyễn MV, Cassar M, Jacquet M, Fiala A, Birman S (2013). A Single Dopamine Pathway Underlies Progressive Locomotor Deficits in a Drosophila Model of Parkinson Disease.. Cell Rep.

[18] Martinez VG, Javadi CS, Ngo E, Ngo L, Lagow RD, Zhang B (2007). Age-related changes in climbing behavior and neural circuit physiology in Drosophila.. Dev Neurobiol.

[19] Tanouye MA, Wyman RJ (1980). Motor outputs of giant nerve fiber in Drosophila.. J Neurophysiol.

[20] Allen MJ, Godenschwege TA, Tanouye MA, Phelan P (2006). Making an escape: Development and function of the Drosophila giant fibre system.. Semin Cell Dev Biol.

[21] He Y, Jasper H (2014). Studying aging in Drosophila.. Methods.

[22] Jones MA, Grotewiel M (2011). Drosophila as a model for age-related impairment in locomotor and other behaviors.. Exp Gerontol.

[23] Muñoz-Alarcón A, Pavlovic M, Wismar J, Schmitt B, Eriksson M, Kylsten P, Dushay MS (2007). Characterization of lamin Mutation Phenotypes in Drosophila and Comparison to Human Laminopathies.. PLoS One.

[24] Feany MB, Bender WW (2000). A Drosophila model of Parkinson’s disease.. Nature.

[25] Auluck PK, Bonini NM (2002). Pharmacological prevention of Parkinson disease in Drosophila.. Nat Med.

[26] Trinh K, Moore K, Wes PD, Muchowski PJ, Dey J, Andrews L, Pallanck LJ (2008). Induction of the Phase II Detoxification Pathway Suppresses Neuron Loss in Drosophila Models of Parkinson ’ s Disease.. J Neurosci.

[27] Barone MC, Bohmann D (2013). Assessing neurodegenerative phenotypes in Drosophila dopaminergic neurons by climbing assays and whole brain immunostaining.. J Vis Exp.

[28] Butler EK, Voigt A, Lutz AK, Toegel JP, Gerhardt E, Karsten P, Falkenburger B, Reinartz A, Winklhofer KF, Schulz JB (2012). The mitochondrial chaperone protein TRAP1 mitigates α-Synuclein toxicity.. PLoS Genet.

[29] De Rose F, Marotta R, Talani G, Catelani T, Solari P, Poddighe S, Borghero G, Marrosu F, Sanna E, Kasture S, Acquas E, Liscia A (2017). Differential effects of phytotherapic preparations in the hSOD1 Drosophila melanogaster model of ALS.. Sci Rep.

[30] Khalil B, Cabirol-Pol MJ, Miguel L, Whitworth AJ, Lecourtois M, Liévens JC (2017). Enhancing Mitofusin/Marf ameliorates neuromuscular dysfunction in Drosophila models of TDP-43 proteinopathies.. Neurobiol Aging.

[31] Blagburn JM, Alexopoulos H, Davies JA, Bacon JP (1999). Null mutation in shaking-B eliminates electrical, but not chemical, synapses in the Drosophila giant fiber system: a structural study.. J Comp Neurol.

[32] Speese SD, Ashley J, Jokhi V, Nunnari J, Barria R, Li Y, Ataman B, Koon A, Chang YT, Li Q, Moore MJ, Budnik V (2012). Nuclear envelope budding enables large ribonucleoprotein particle export during synaptic Wnt signaling.. Cell.

[33] Ganetzky B, Flanagan JR (1978). On the relationship between senescence and age-related changes in two wild-type strains of Drosophila melanogaster.. Exp Gerontol.

[34] Le Bourg E, Lints FA (1992). Hypergravity and aging in Drosophila melanogaster. 4. Climbing activity.. Gerontology.

[35] Wu JS, Luo L (2006). A protocol for dissecting Drosophila melanogaster brains for live imaging or immunostaining.. Nat Protoc.

