# Supplementary material for: Neuronal Lamin regulates motor circuit integrity and controls motor function and lifespan
Source: Cell Stress. 2018 Aug 17;2(9):225–32. doi: 10.15698/cst2018.09.152 (PMC6558924; doi:10.15698/cst2018.09.152)
Supplement: Supplementary file 1 [file ces-02-225-s01.pdf]

SUPPLEMENTARY FIGURES

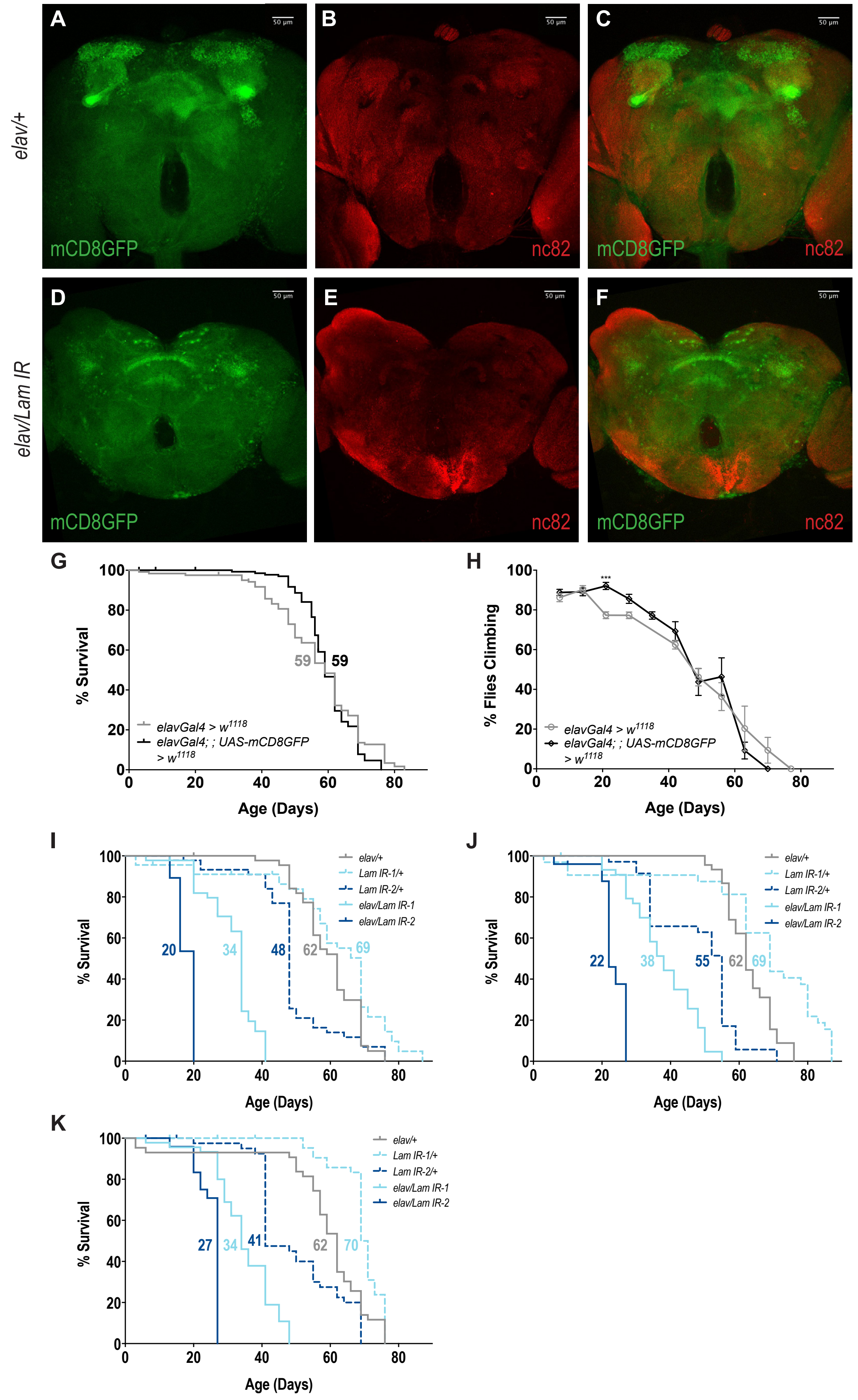

Fig. S1 Neuronal *Lamin* knockdown causes decrease in lifespan with no overt change in brain morphology. A Representative image of *elav/+* depicting mCD8GFP expression, **B** nc82 staining, **C** and merge showing similar coverage. **D** Representative image of *elav/Lam IR* showing mCD8GFP expression, **E** nc82 staining **F** and merge showing similar coverage and no overt morphological changes with *Lamin* knockdown. Control lines (*elavGal4; +/+; UAS-mCD8GFP*) used in this study show no difference in **G** lifespan **H** or climbing compared to *elavGal4* alone. n≥90 animals per group. **I-K** Individual replicates for lifespan experiments show a consistent decrease in *elav/Lam IR* lifespan compared to controls. n≥25 animals per group. Data are mean ± SEM. Climbing was analysed using multiple t-tests with Holdam-Sidak correction for multiple comparisons. Lifespan analysis was done using a log-rank (Mantel-Cox test). ***p<0.001. *elav/+ = elavGal4; +/+; UAS-mCD8GFP* > w^{1118}*; Lam IR/+ =* w^{1118} *> Lam IR; elav/Lam IR = elavGal4; +/+; UAS-mCD8GFP > Lam IR.*

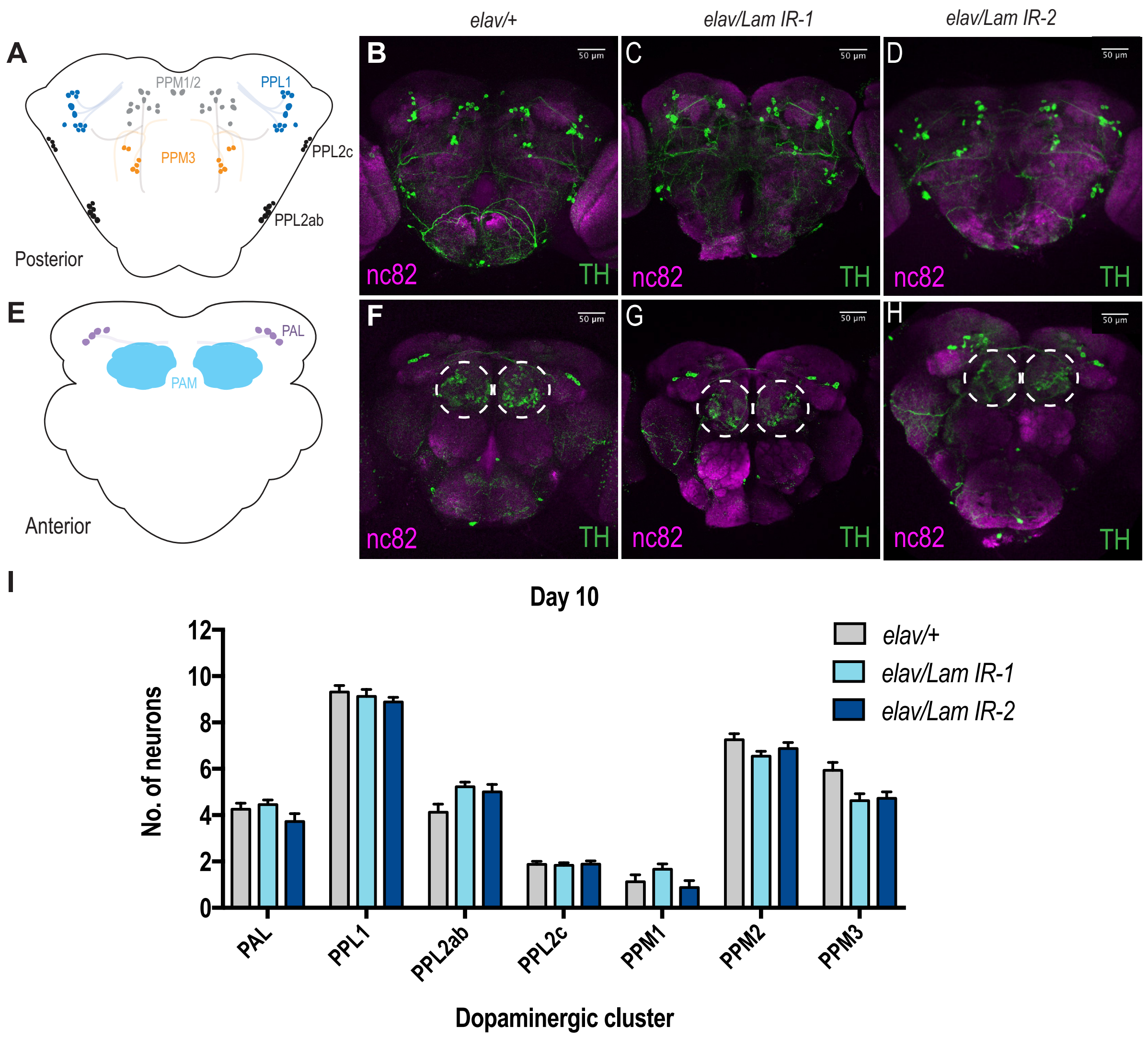

Fig. S2 *Lamin* knockdown has no effect on other dopaminergic neuron clusters in young flies. A Schematic of the posterior dopaminergic neurons of the *Drosophila* brain. Representative images of posterior dopaminergic neuron clusters in **B** *elav/+* and **C-D** *Lamin* knockdown (*elav/lam IR*) flies at 10 days old. **E** Schematic of the anterior dopaminergic neurons of the *Drosophila* brain. Representative images of the anterior dopaminergic neuron clusters in **F** *elav/+* and **G-H** 10-day old *Lamin* knockdown flies (*elav/lam IR*). **I** Neuronal *Lamin* knockdown (*elav/lam IR*) causes no significant difference in dopaminergic neuron number in the posterior clusters of the brain of 10-day old flies. n≥10 animals per group. Data are mean ± SEM. Data was analysed using student's *t*-test. *elav/+ = elavGal4; +/+; UAS-mCD8GFP >* w^{1118}*; elav/Lam IR = elavGal4; +/+; UAS-mCD8GFP > Lam IR.*

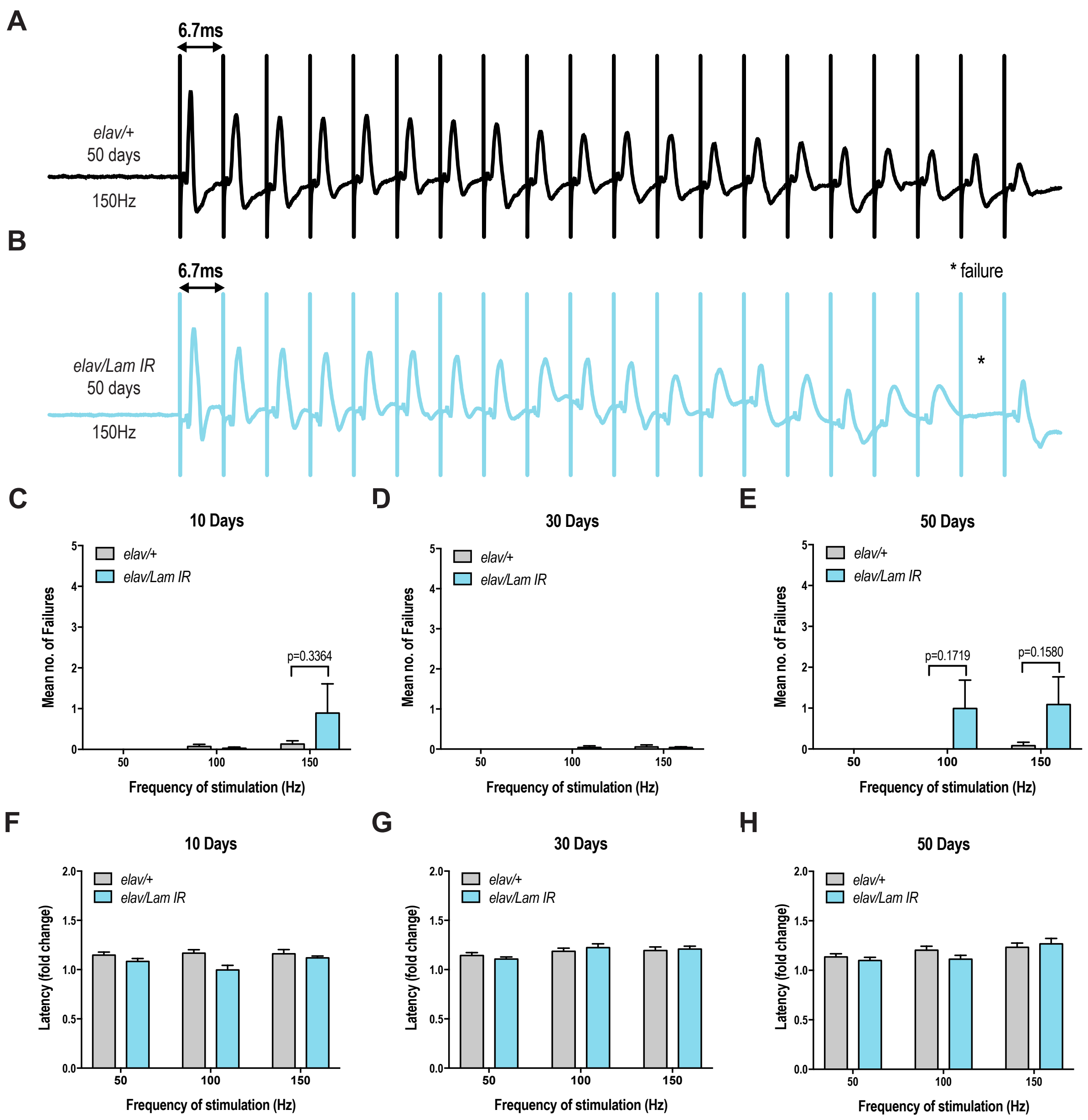

Fig. S3 *Lamin* knockdown has no significant effect on TTM response. Example traces from the TTM in **A** *elav/+* and **B** *Lamin* knockdown (*elav/Lam IR*) flies aged 50 days, stimulated at 150 Hz. Asterisks indicate failure to respond. Mean number of failures for *elav/+* and *Lamin* knockdown flies (*elav/Lam IR*) at **C** 10 days, **D** 30 days and **E** 50 days old. **F-H** No significant difference in TTM latency was found with *Lamin* knockdown (*elav/Lam IR*) at any age, when compared to *elav/+*. n≥7 animals per group. Data are mean ± SEM. Data was analysed using two-way ANOVA with post hoc Bonferroni correction for multiple comparisons. Response latency was analysed using multiple t-tests with Holdam-Sidak correction for multiple comparisons. *elav/+* = *elavGal4; +/+; UAS-mCD8GFP* > *w^{1118}; elav/Lam IR* = *elavGal4; +/+; UAS-mCD8GFP* > *Lam IR.*
